# Heterogeneous vascular response after implantation of bare nitinol self-expanding stents in the swine femoropopliteal artery

**DOI:** 10.1007/s12928-022-00889-5

**Published:** 2022-10-18

**Authors:** Osami Kawarada, Fumiyuki Otsuka, Kojiro Miki, Masayasu Ikutomi, Kozo Okada, Soshiro Ogata, Kunihiro Nishimura, Peter J. Fitzgerald, Yasuhiro Honda

**Affiliations:** 1 Department of Cardiovascular Medicine, National Cerebral and Cardiovascular Center, 6-1 Kishibe-Shimmachi, Suita city, Osaka 564-8565 Japan; 2 Kawarada Cardio Foot Vascular Clinic, Osaka, Japan; 3 Division of Cardiovascular Medicine, Stanford University School of Medicine, Stanford, CA USA; 4 Department of Preventive Medicine and Epidemiology, Center for Cerebral and Cardiovascular Disease Information, National Cerebral and Cardiovascular Center, Osaka, Japan

**Keywords:** Femoropopliteal intervention, Stent, Angiography, Histology, Necropsy study

## Abstract

**Background:**

Mechanism of femoropopliteal in-stent restenosis has been underappreciated.

**Aim:**

The aim of this animal study was to elucidate vascular response after femoropopliteal bare nitinol self-expanding stents (SESs) implantation.

**Methods:**

Misago, Smart Flex, or Innova stent was randomly implanted in 36 swine femoropopliteal arteries. At week 4, quantitative vessel analysis (QVA) was performed on 36 legs, of which 18 underwent histological evaluation after angiography. The remaining 18 legs underwent QVA and histological evaluation at week 13.

**Results:**

Fibrin deposition was excessive at week 4. Internal elastic lamina (IEL) progressively enlarged over time, and vessel injury developed from mild level at week 4 to moderate level at week 13. Vessel inflammatory reaction was mild to moderate at week 4, and was moderate to severe at week 13. Increased fibrin deposition was an early-acting, IEL enlargement and increased vessel inflammation were long-acting, and increased vessel injury and giant cells infiltration were late-acting contributors to neointimal hyperplasia (NIH). Stent type altered time-dependent process of vessel injury, vessel inflammation, eosinophils and giant cells infiltration. Misago had less fibrin deposition and vessel enlargement, and less progressive vessel injury, vessel inflammation, and eosinophils and giant cells infiltration. Net lumen as assessed by percent diameter stenosis or minimum lumen diameter was preserved with Misago, but was not preserved with the other stents.

**Conclusions:**

In the context of bare nitinol SES platform with less progressive mechanical stress and inflammatory reaction, the advantage of less NIH outweighed the disadvantage of less vessel enlargement, leading to net lumen preservation.

**Supplementary Information:**

The online version contains supplementary material available at 10.1007/s12928-022-00889-5.

## Introduction

A late catch-up phenomenon due to neointimal hyperplasia (NIH) poses a challenge to femoropopliteal drug-eluting stenting [1–3]. In the first place, the situation surrounding femoropopliteal stenting is exceptional from the perspectives of anatomical physiology and endovascular therapeutics: (1) the femoropopliteal artery can be exposed to strong external physical forces such as compression, elongation, and torsion, causing distinctive in-stent shear stress and stent-related vessel trauma; (2) the femoropopliteal artery is much more elastic than coronary arteries, causing steady vessel constraint; and (3) once nitinol self-expanding stents (SESs) deployed, it can store more strain energy than balloon-expandable stents (BESs) made of stainless steel or cobalt alloy implanted in a coronary artery, and exert a continuous force upon the vessel wall, causing progressive stent expansion and vessel enlargement [4–6]. Therefore, vascular response to femoropopliteal stenting might be more dynamic than those to coronary artery counterparts.

Nevertheless, the mechanism of in-stent restenosis of bare nitinol SES platforms per se still remains unclear, which impedes the development of appropriate femoropopliteal DES platforms. Although there are a few necropsy studies comparing 2 types of femoropopliteal paclitaxel-eluting stents [7, 8], the differences of bare nitinol SES platforms still have been underestimated. Currently, the difference in formation of NIH after implantation of bare nitinol SESs with high versus low chronic outward force is under investigation in a randomized controlled clinical trial [9]. The aim of this animal study was to elucidate vascular response and neointimal process after implantation of 3 bare nitinol SESs under identical conditions. For this purpose, swine femoropopliteal arteries implanted with representative bare nitinol SESs such as Misago (Terumo Corporation, Tokyo, Japan), Smart Flex (Cordis, Cardinal Health Corporation, Fremont, CA), or Innova (Boston Scientific, Natick, MA) were examined with a direct side-by-side comparison using quantitative vessel analysis (QVA) and histology.

## Methods

A total of 36 legs in 20 healthy swine without any lesions, weighing 67.6–75.0 kg were used in this animal study (Supplemental Fig. 1). All swine received aspirin (81 mg/day) and clopidogrel (75 mg/day) orally 3 days before catheterization with continued dosing throughout the study. All swine were sedated intramuscular midazolam (0.2 mg/kg) and medetomidine (0.04 mg/kg), and then intubated for mechanical ventilation. Inhalation of 2–4% sevoflurane was used for the maintenance of general anesthesia throughout the procedure. Arterial access was obtained by deploying an arterial sheath in the common carotid artery. Intravenous heparin (300 U/kg) was administered before catheterization. The type of 6 mm × 60 mm bare nitinol SES (Misago, Smart Flex, or Innova) was randomly chosen to be implanted in the distal femoropopliteal artery (proximal reference diameter of stented segment: 4.9–5.1 mm, distal reference diameter of stented segment: 3.6–4.0 mm, mean reference diameter of stented segment: 3.7–4.0 mm). Neither predilatation nor postdilatation was performed. At week 4, angiographic evaluation was performed in 36 legs, of which 18 underwent histological evaluation after angiography. The remaining 18 legs underwent angiographic and histological evaluation at week 13 (Supplemental Fig. 2). The 3 bare nitinol SESs have distinctly different designs. The Misago stent has an open-cell design with 8 zigzag cells and 2 links. The Smart Flex stent has a fully connected design with helical strut bands and interconnecting flex bridges. The Innova stent has an open-cell design with a peak-to-valley structure. All procedures involving animals were approved by the institutional animal care and use committee.

### Quantitative vessel analysis

QVA was performed with QAngio XA 7.3 (Medis Medical Imaging Systems, Leiden, Netherlands) at an independent core laboratory (Stanford Cardiovascular Core Analysis Laboratory, Stanford, CA) that was blinded to procedural details and histological results. The outer diameter of the contrast-filled catheter was used as the calibration standard. Minimum lumen diameter (MLD) was measured from the single worst view. Percent diameter stenosis (%DS) was calculated based on MLD and the interpolated reference vessel diameter.

### Histological analysis

Histological specimens were prepared by an independent pathology laboratory (Alizée Pathology, Thurmont, MD). After the animals were euthanized, perfusion fixation of the stented arteries was performed. The stented arteries were embedded in methyl methacrylate and sawed serially at equally spaced intervals to obtain 6 sections from each stent. In the segmental analysis, the 6 sections of each stent were divided into the proximal, mid, and distal portions, with each portion containing 2 sections. All sections were stained hematoxylin and eosin and elastin trichrome. Histomorphometric analysis was independently performed by an experienced investigator (F.O.) in the National Cerebral and Cardiovascular Center Biobank. Vessel injury was scored according to the method of Schwartz et al. [10]; score 0 corresponded to intact internal elastic lamina (IEL), typically denuded endothelium, and compressed but not lacerated media; score 1 corresponded to lacerated and typically compressed but not lacerated media; score 2 corresponded to lacerated IEL, visibly lacerated media, and intact but compressed external elastic lamina (EEL); and score 3 corresponded to lacerated EEL, typically large lacerations in the media extending through the EEL, and sometimes with coil wires residing in the adventitia. The average injury score for each segment was calculated by dividing the sum of injury scores by the total number of struts in the examined section. Vessel inflammation was semiquantitatively scored for each section, as previously described [11]; score 0 corresponded to ≤ 25% of struts with < 10 inflammatory cells. score 1 corresponded to ≤ 25% of struts with ≥ 10 inflammatory cells; score 2 corresponded to 25–50% of struts with ≥ 10 inflammatory cells; score 3 corresponded to ≥ 50% of struts with ≥ 10 inflammatory cells; and score 4 corresponded to ≥ 2 strut-associated granulomatous inflammatory reactions. The amount of fibrin deposition and the number of eosinophils and giant cells around stent struts was each expressed as a percentage of the total number of struts in each section.

### Statistical analysis

Continuous variables were presented as means and 95% confidence intervals. QVA data were analyzed using a mixed model assuming a compound symmetry covariance structure. Stent type, section site, time point, or segment site was modeled as fixed effects. In the models, random intercepts were modeled by animal ID. Multiple comparisons of estimated marginal means were performed based on post hoc analyses of linear mixed models with Bonferroni corrections. Histological data at 2 time points were assessed using linear mixed models with random intercept for animals. Relationships between histological variables and neointimal area were also assessed using linear mixed models in which histological variables were modeled as fixed effects and random intercepts were modeled by animal ID. The differences were considered significant when *P* values were < 0.05. SPSS version 22 (IBM Corp., Armonk, NY, USA) was used for all analyses.

## Results

### Quantitative vessel analysis

**Fig. 1:**
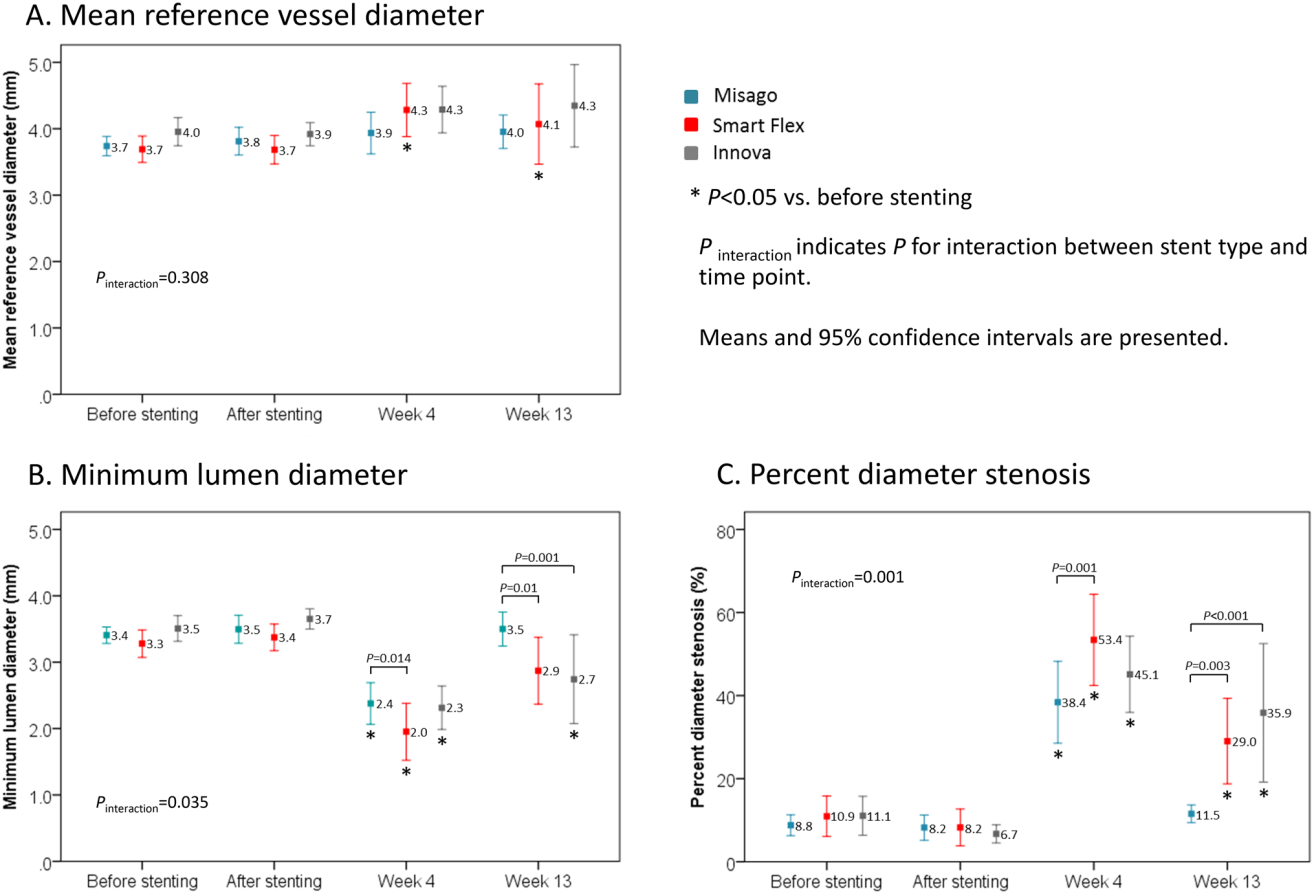
Quantitative vessel analysis

QVA results for each time point are shown in Fig. 1. Mean reference vessel diameter (RVD), MLD and %DS before stenting were comparable among all stents (*P* = 0.34, 0.385, and 0.831, respectively). An interaction effect between stent type and time point was observed in MLD and %DS (*P*_interaction_ = 0.035 and 0.001, respectively); at week 4, the 3 stents showed smaller MLD and higher %DS as compared with before stenting (all *P* < 0.001); at week 13, Misago revealed a recovery of MLD and %DS to a level of before stenting, while Smart Flex had higher %DS (*P* = 0.002) and Innova had smaller MLD (*P* = 0.001) and higher %DS (*P* < 0.001) compared to before stenting. Also, only Smart flex had larger mean RVD at weeks 4 and 13 than before stenting.

### Histological analysis

**Fig. 2:**
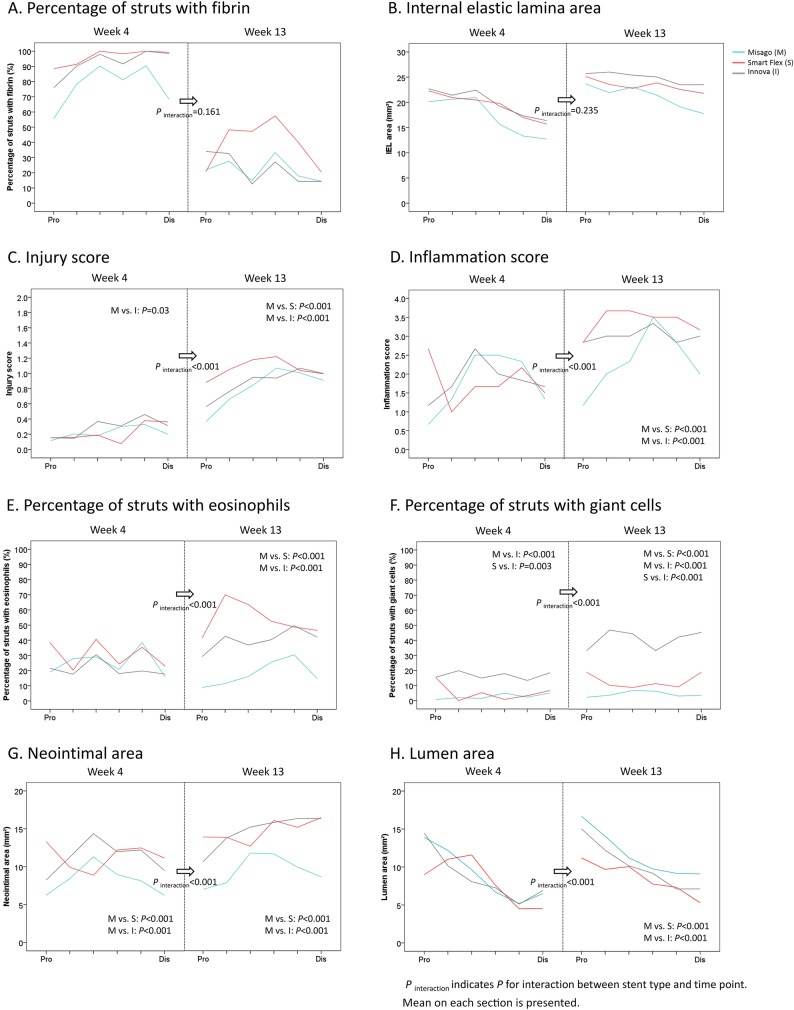
Section-based distribution of histological variables

**Fig. 3:**
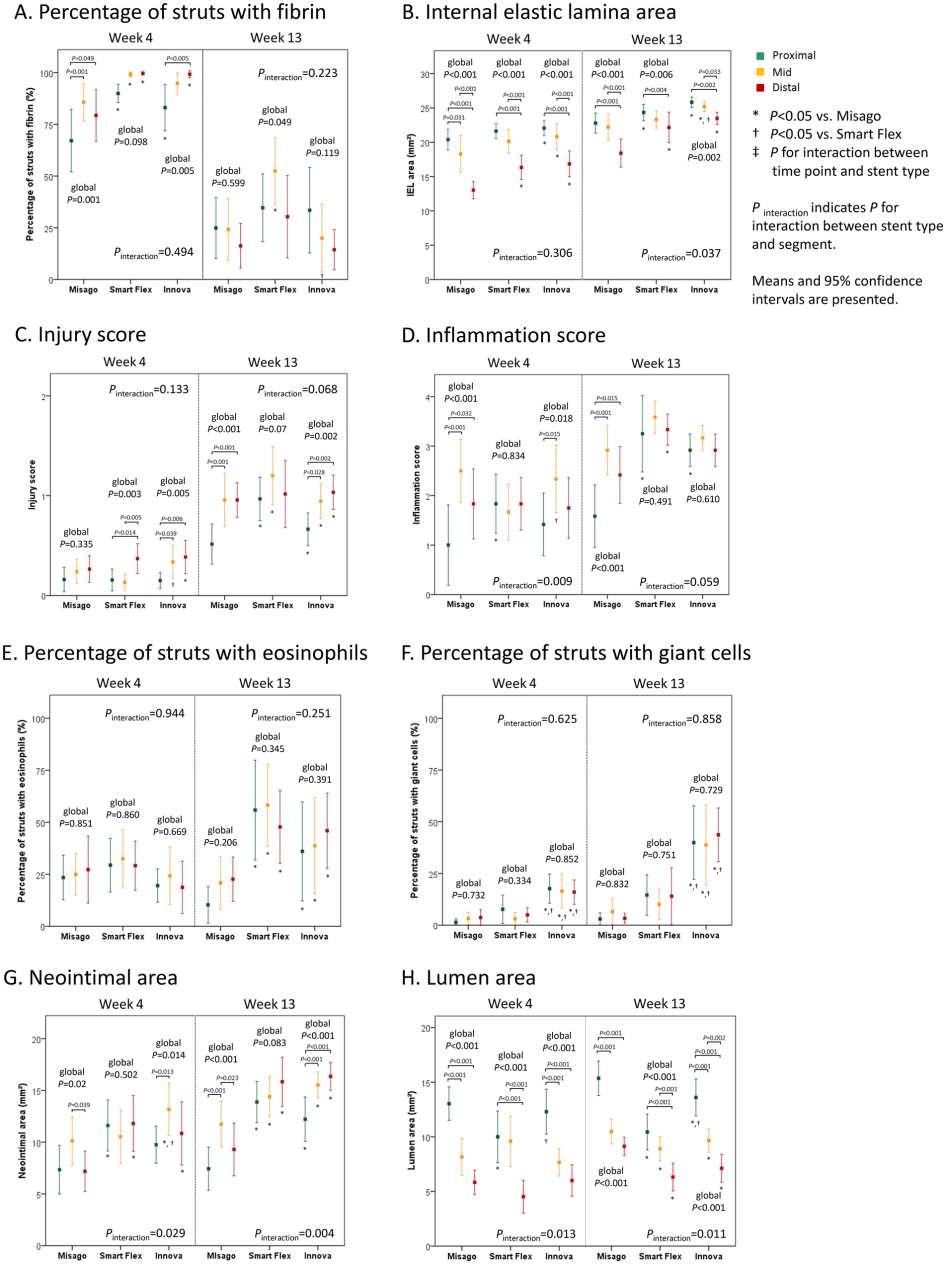
Segment-based analysis of histological variables

Histological data for each time point are summarized in Table 1. Representative histological images are shown in Supplemental Fig. 3. Section-based distributions of histological variables are shown in Fig. 2. Segment-based analyses of histological variables are shown in Fig. 3.

**Table 1:** Histological factors at weeks 4 and 13

	Misago	Smart Flex	Innova	*P* value
Percentage of struts with fibrin (%)				
Week 4	77.4 (70.4–84.4)	96.3 (94.2–98.3)*	92.4 (88.0–96.8)*	<0.001
Week 13	21.8 (14.6–29.0)	39.1 (29.5–48.8)*	22.6 (13.8–31.4)†	0.002
* P* value	<0.001	<0.001	<0.001	
IEL area (mm^2^)				
Week 4	17.2 (15.8–18.7)	19.4 (18.2–20.5)*	19.9 (18.8–21.1)*,†	<0.001
Week 13	21.1 (20.0–22.3)	23.3 (22.4–24.2)*	24.8 (24.3–25.4)*,†	<0.001
* P* value	0.001	<0.001	<0.001	
Injury score				
Week 4	0.2 (0.2–0.3)	0.2 (0.1–0.3)	0.3 (0.2–0.4)*	0.025
Week 13	0.8 (0.7–0.9)	1.1 (0.9–1.2)*	0.9 (0.8–1.0)*	<0.001
* P* value	0.001	<0.001	<0.001	
Inflammation score				
Week 4	1.8 (1.4–2.2)	1.8 (1.5–2.1)	1.8 (1.5–2.2)	0.059
Week 13	2.3 (2.0–2.7)	3.4 (3.1–3.7)*	3.0 (2.8–3.2)*	<0.001
* P *value	0.077	<0.001	0.001	
Percentage of struts with eosinophils (%)				
Week 4	25.2 (18.7–31.7)	30.3 (23.6–37.0)	20.8 (14.7–27.0)	0.062
Week 13	17.9 (12.0–23.9)	53.9 (43.2–64.7)*	40.2 (28.8–51.6)*	<0.001
* P* value	0.064	0.063	0.02	
Percentage of struts with giant cells (%)				
Week 4	2.8 (1.2–4.3)	5.2 (2.7–7.8)	16.7 (13.0–20.4)*,†	<0.001
Week 13	4.3 (2.1–6.5)	12.9 (7.3–18.4)*	40.8 (32.0–49.6)*,†	<0.001
* P* value	0.681	0.113	<0.001	
Neointimal area (mm^2^)				
Week 4	8.2 (7.0–9.5)	11.3 (10.0–12.7)*	11.3 (9.9–12.6)*	<0.001
Week 13	9.5 (8.2–10.8)	14.7 (13.6–15.8)*	14.7 (13.7–15.7)*	<0.001
* P* value	0.934	0.015	0.004	
Lumen area (mm^2^)				
Week 4	9.0 (7.7–10.3)	8.0 (6.7–9.4)	8.7 (7.4–9.9)	0.033
Week 13	11.7 (10.5–12.8)	8.6 (7.6–9.5)*	10.1 (9.0–11.3)*	<0.001
* P* value	<0.001	0.266	0.617	

Values are presented as means (95% confidence intervals)

*P<0.05 vs. Misago, † *P*<0.05 vs. Smart Flex

### Fibrin deposition

At week 4, fibrin deposition was generally excessive (the percentage of struts with fibrin: 77.4–96.3%), and the percentage of struts with fibrin was lower with Misago compared with the other stents (Misago vs. Smart Flex: *P* < 0.001; Misago vs. Innova: *P* = 0.007). At week 13, the percentage with the 3 stents decreased to moderate level (21.8–39.1%) (all *P* < 0.001), and was higher with Smart Flex than with the other stents (Misago vs. Smart Flex: *P* = 0.004; Smart Flex vs. Innova: *P* = 0.017) (Table 1, Fig. 2A). With regard to fibrin distribution, the proximal segment mostly had lower percentage than the other segments at week 4, while the percentage was similar across all segments at week 13 (Fig. 3A).

### Internal elastic lamina enlargement

At week 4, Misago had the smallest internal elastic lamina (IEL) area and Innova had the largest IEL area among the 3 stents (all *P* < 0.001). With the 3 stents, IEL area increased from weeks 4 to 13 (Misago: *P* = 0.001, Smart Flex: *P* < 0.001, Innova: *P* < 0.001). At week 13 as well, Misago had the smallest IEL area and Innova had the largest IEL area among the 3 stents (Misago vs. Smart Flex: *P* < 0.001, Misago vs. Innova: *P* < 0.001, Smart Flex vs. Innova: *P* = 0.004) (Table 1, Fig. 2B). With regard to IEL distribution, taperedness of IEL distribution was similar among the 3 stents at week 4 (*P*_interaction_ = 0.306), but at week 13 was more tapered with Misago compared to the other stents (*P*_interaction_ = 0.037) (Fig. 3B).

### Vessel injury

There was an interaction effect of stent type on the association between injury score and time point (*P*_interaction_ < 0.001) (Fig. 2C); at week 4, vessel injury was generally mild (injury score: 0.2–0.3); at week 13, vessel injury with the 3 stents increased to moderate level (injury score: 0.8–1.1) (Misago, *P* = 0.001; Smart Flex, *P* < 0.001; Innova, *P* < 0.001); consequently, injury score at week 13 was lower with Misago than with the other stents (each *P* < 0.001) (Table 1, Fig. 2C). With regard to vessel injury distribution, gradual increase in injury score from proximal to distal segments was similar among the 3 stents both at weeks 4 and 13 (*P*_interaction_ = 0.133 and *P*_interaction_ = 0.068, respectively) (Fig. 3C).

### Vessel inflammation

There was an interaction effect of stent type on the association between inflammation score and time point (*P*_interaction_ < 0.001) (Fig. 2D); at week 4, vessel inflammation was generally moderate (all inflammation scores: 1.8); at week 13, inflammation score increased to 3.4 with Smart Flex (*P* < 0.001) and 3.0 with Innova (*P* = 0.001), and was on the increase to 2.3 with Misago (*P* = 0.077); consequently, inflammation score at week 13 was lower with Misago than with the other stents (each *P* < 0.001) (Table 1, Fig. 2D). Also, there was an interaction effect of stent type on the segmental distribution of vessel inflammation at weeks 4 and 13 (*P*_interaction_ = 0.009 and 0.059, respectively); inflammation score with Misago was lower in the proximal segment than in the other segments at weeks 4 and 13 (each global *P*_<_0.001), while inflammation scores with Smart Flex and Innova were equivalent among all segments at week 13 (global *P* = 0.491 and 0.610, respectively) (Fig. 3D).

### Eosinophils infiltration

Stent type had an interaction effect on the association between the percentage of struts with eosinophils and time point (*P*_interaction_ < 0.001) (Fig. 2E); at week 4, eosinophils infiltration was generally moderate (the percentage of struts with eosinophils: 20.8–30.3%); at week 13, the percentage increased to 40.2% with Innova (*P* = 0.02), and was on the increase to 53.9% with Smart Flex (*P* = 0.063), whereas on the decrease to 17.9% with Misago (*P* = 0.064); consequently, the percentage at week 13 was lower with Misago compared with the other stents (each *P* < 0.001) (Table 1, Fig. 2E**)**. At weeks 4 and 13, the distributions of eosinophils infiltration were equivalent among all segments with the 3 stents (Fig. 3E).

### Giant cells infiltration

Stent type had an interaction effect on the association between the percentage of struts with giant cells and time point (*P*_interaction_ < 0.001) (Fig. 2F); at week 4, giant cells infiltration was more marked with Innova than Misago (*P* < 0.001) and Smart Flex (*P* = 0.003) (the percentage of struts with giant cells: Misago 2.8%, Smart Flex 5.2%, and Innova 16.7%); at week 13, only with Innova the percentage increased to severe level (*P* < 0.001), whereas the percentage with Misago (*P* = 0.681) and Smart Flex (*P* = 0.113) remained unchanged (the percentage: Misago 4.3%, Smart Flex 12.9%, and Innova 40.8%); consequently, Misago exhibited the lowest percentage and Innova exhibited the highest percentage among the 3 stents (all *P* < 0.001) (Table 1, Fig. 2F). At weeks 4 and 13, the distributions of giant cells infiltration were equivalent among all segments with the 3 stents (Fig. 3F).

### Neointimal hyperplasia

As with vessel injury, inflammation, and eosinophils and giant cells infiltration, stent type had an interaction effect on the neointimal growth; at week 4, neointimal area was smaller with Misago than the other stents (each *P* < 0.001).; at week 13, neointimal area increased with Smart Flex and Innova (*P* = 0.015 and 0.004, respectively), but remained unchanged with Misago (*P* = 0.934); consequently, neointimal area at week 13 was smaller with Misago than with the other stents (each *P* < 0.001) (Table 1, Fig. 2G). Also, neointimal distribution was heterogeneous among the 3 stents both at weeks 4 (*P*_interaction_ = 0.029) and 13 (*P*_interaction_ = 0.004); in particular at week 13, Misago had larger neointimal area in the mid segment than in the other segments (global *P* < 0.001), while Smart Flex and Innova had larger neointimal area in the distal segments than in the other segments (global *P* = 0.083 and *P* < 0.001, respectively) (Fig. 3G).

### Histological factors associated with neointima

**Fig. 4:**
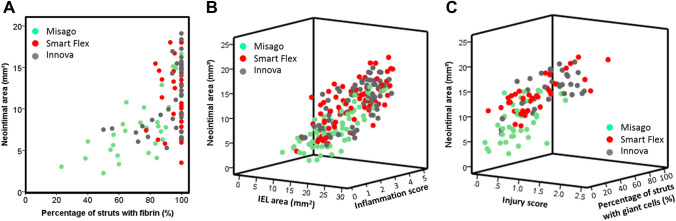
Color scatter plot representing the time-dependent relationship between histological factors and NIH. **A** Relationship between the percentage of struts with fibrin and neointimal area at week 4. Stent type-based clustering indicates less fibrin deposition and less NIH with Misago. **B** Relationship between IEL area, inflammation score and neointimal area at weeks 4 and 13. Stent type-based clustering indicates less IEL enlargement, less vessel inflammation, and less NIH with Misago. **C** Relationship between injury score, the percentage of struts with giant cells, and neointimal area at week 13. Stent type-based clustering indicates less vessel injury, less giant cells infiltration, and less NIH with Misago

Relationships between neointimal area and histological variables are shown in Table 2. At week 4, according to multivariate analysis following univariate analysis, IEL area, percentage of struts with fibrin, and inflammation score were positively associated with neointimal area at week 4. At week 13, results of the univariate analysis were similar to those for week 4 except for the percentage of struts with fibrin. Multivariate analysis revealed that IEL area, injury score, inflammation score, and percentage of struts with giant cells were positively associated with neointimal area at week 13. Color scatter plots of these significant variables are shown in Fig. 4, representing stent type-based clustering.

**Table 2:** Histological factors associated with neointimal area

	Univariate				Multivariate			
	Regression coefficient	*P *value	95% confidence intervals		Regression coefficient	*P *value	95% confidence intervals	
IEL area	0.413	<0.001	0.244	0.582	0.358	<0.001	0.229	0.486
Injury score	6.303	<0.001	3.402	9.204	2.347	0.061	− 0.106	4.8
Percentage of struts with fibrin	0.11	<0.001	0.075	0.146	0.07	<0.001	0.041	0.099
Inflammation score	2.22	<0.001	1.646	2.794	1.058	0.001	0.441	1.675
Percentage of struts with eosinophils	0.104	<0.001	0.07	0.138	0.025	0.112	− 0.006	0.056
Percentage of struts with giant cells	0.113	0.001	0.047	0.178	0.008	0.753	− 0.042	0.058

	Univariate				Multivariate			
	Regression coefficient	*P *value	95% confidence intervals		Regression coefficient	*P *value	95% confidence intervals	
IEL area	0.833	<0.001	0.612	1.054	0.417	<0.001	0.235	0.6
Injury score	6.216	<0.001	4.469	7.963	3.115	<0.001	1.681	4.548
Percentage of struts with fibrin	0.006	0.699	-0.025	0.037				
Inflammation score	2.812	<0.001	2.154	3.471	1.41	<0.001	0.762	2.059
Percentage of struts with eosinophils	0.091	<0.001	0.072	0.11	0.005	0.625	− 0.016	0.027
Percentage of struts with giant cells	0.102	<0.001	0.074	0.131	0.048	<0.001	0.024	0.071

*IEL* internal elastic lamina

### Lumen transition and distribution

**Fig. 5:**
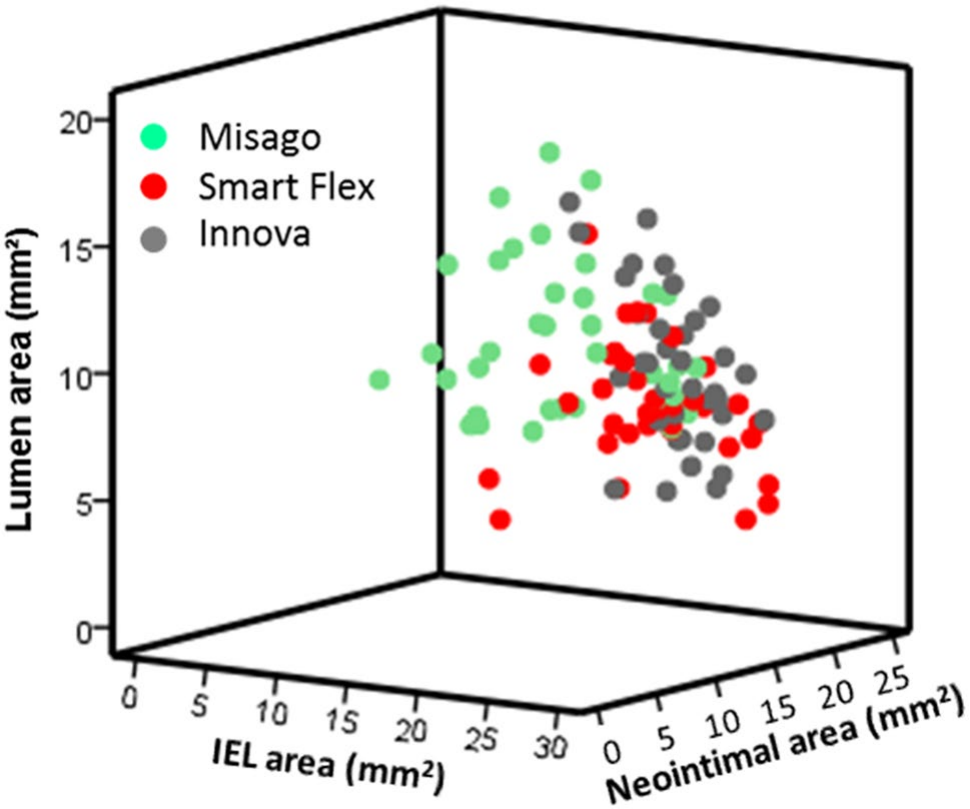
Color scatter plot representing the relationship between IEL area, neointimal area, and resultant lumen area at week 13. Stent type-based clustering indicates larger lumen area with Misago because of smaller areas of IEL and neointima compared to the other stents

**Fig. 6:**
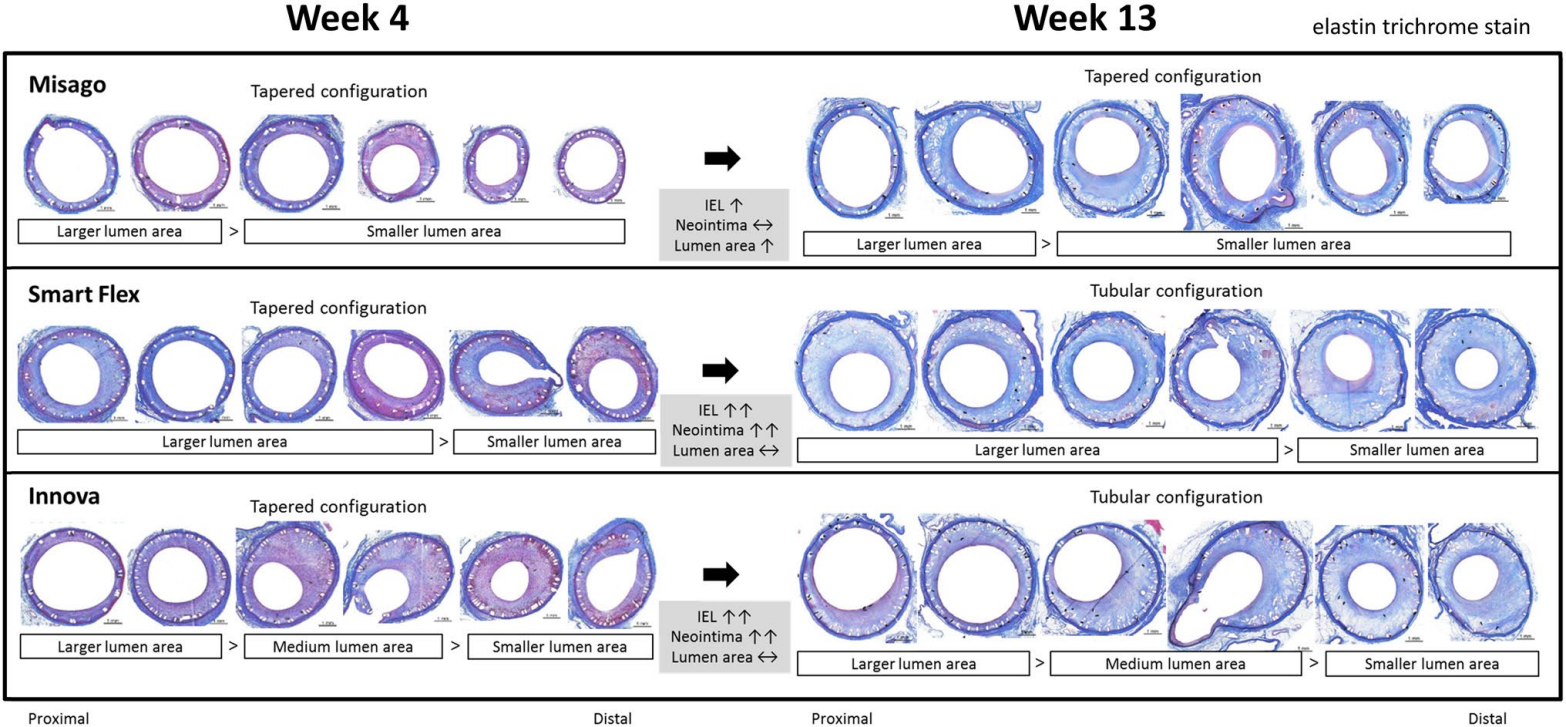
Representative cross-sections of stents at weeks 4 and 13. With Misago, the trade-off between progressively enlarged IEL and unchanged NIH led to increase in lumen area. On the other hand, with Smart Flex and Innova, progressively increased NIH offset the delayed expanding space created by more progressive enlargement of IEL, leading to no increase in lumen area. Also, lumen area distribution of each stent was unique; Misago had larger lumen area in the proximal segment, Smart Flex had smaller lumen area in the distal segment, and Innova had larger lumen area in the proximal segment, medium lumen area in the mid segment, and smaller lumen area in the distal segment. elastin trichrome stain

In line with NIH, stent type had an interaction effect on lumen loss; although lumen area at week 4 was comparable among the 3 stents, lumen area with Misago increased at week 13 as compared to that at week 4 (*P* < 0.001) and remained unchanged with Smart Flex and Innova (*P* = 0.266 and 0.617, respectively); consequently, at week 13 Misago exhibited larger lumen area than the other stents (each *P* < 0.001) (Table 1, Fig. 2H). Also, stent type had an interaction effect on the association between lumen area and segment at weeks 4 and 13 (*P*_interaction_ = 0.013 and 0.011, respectively); lumen area was larger in the proximal segment with Misago and Innova, and smaller in the distal segment with Smart Flex and Innova (all global *P* < 0.001) (Fig. 3H). Given that lumen area can be figured out by subtracting neointimal area from IEL area, color scatter plots of these variables at week 13 are shown in Fig. 5, representing stent type-based clustering. Representative cross-sections of stents implanted for vascular remodeling and neointimal process are shown in Fig. 6.

## Discussion

**Fig. 7:**
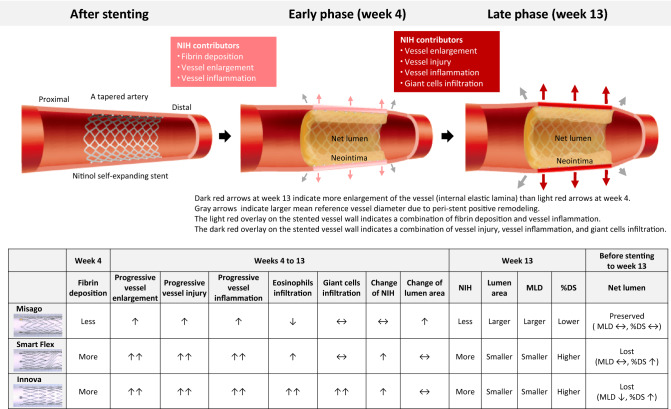
Vascular response after bare nitinol self-expanding stenting in the femoropopliteal artery. Upper: schema of overall vascular response to implantation of bare nitinol self-expanding stent and histological contributors to neointimal hyperplasia. Lower: comparison of angiographical and histological findings among the 3 stents. Note that net lumen was preserved with Misago, but lost with the other stents

The main findings of this study are as follows: (1) net lumen such as MLD and %DS was preserved only with Misago, but was not preserved with the other stents; (2) only Smart Flex had larger mean RVD over time than before stenting; (3) the degree of fibrin deposition, vessel enlargement, injury, and inflammatory reaction was lower with Misago than with the other stents; (4) an interaction effect between stent type and time point was observed in vessel injury, vessel inflammation, eosinophils infiltration, giant cells infiltration, NIH, and lumen; (5) Misago had lower neointimal area and larger lumen area than the other stents at week 13; (6) stent type also had an interaction effect on the distributions of IEL, vessel inflammation, NIH, and lumen at weeks 4 or 13; and (7) increased fibrin deposition was an early-acting, progressive IEL enlargement and vessel inflammation were long-acting, and progressive vessel injury and giant cells infiltration were late-acting contributors to NIH (Fig. 7).

### Neointimal proliferation and distribution

In the swine coronary arteries implanted with bare BESs, neointima peaks at week 4, and thereafter regresses [12]. However, the present study found that neointima does not regress even after week 4, and stent type affects the degree of neointimal growth (Fig. 2G); Misago maintained neointimal area at a constant level, while the other stents increased neointimal area over time. These findings indicate that peak time of neointimal growth after femoropopliteal stenting is more delayed than that after coronary stenting. The present study also found that neointima is more likely to grow in the mid segment with Misago and in the distal segment with the other stents at week 13 (Fig. 3G). These findings indicate heterogeneous NIH distribution even among bare nitinol SESs.

### Hallmark of fibrin deposition

Fibrin deposition around stent struts that is a marker of mast cell activation around biomaterial implants in an early stage of inflammatory reaction, is followed by a neointimal healing process, when smooth muscle cells and the extracellular matrix are substituted for fibrin [12–15]. In the swine coronary arteries implanted with bare BESs, the percentage of struts with fibrin at week 4 is only approximately 10% [16], and fibrin deposition is correlated with NIH [17]. The present study found that the percentage of struts with fibrin at week 4 is very high as compared with previous coronary findings, and Misago has the lowest percentage among the 3 stents (Misago: 77.4%, Smart Flex: 96.3%, Innova: 92.4%). Also, the present study found that increased percentage of struts with fibrin is independently associated with neointimal area at week 4. These findings indicate that fibrin deposition after femoropopliteal stenting is more abundant than that after coronary stenting, and fibrin deposition is an early-acting contributor to NIH. Therefore, suppression of fibrin deposition is an initial window of opportunity for minimizing NIH.

### Hallmark of vessel enlargement and injury

Coronary bare BESs instantly can cause the vessel enlargement and coexistent vessel injury (injury score of less than 1.0), which is sustained at a constantly low level [15, 18, 19]. In the present study, 6 mm diameter stent was implanted in the artery with mean RVD of approximately 4 mm (3.7–4.0 mm), suggesting stent/vessel diameter ratio of 1.5. Reflecting the characteristics of SES, the 3 stents enlarged IEL over time with the smallest IEL area in Misago. Also, the present study found that only Smart Flex had larger mean RVD over time as compared to before stenting, suggesting Smart Flex-specific increasing stent edge stress. Intriguingly, although the 3 stents exhibited a similar longitudinal taperedness of IEL at week 4, stent type affected the taperedness at week 13 (*P*_interaction_ = 0.037); Misago maintained tapered vessel configuration, whereas the other stents had less tapered vessel configuration (Fig. 3B). On the other hand, vessel injury developed from mild level (injury score of 0.2–0.3) at week 4 to moderate level (injury score of 0.8–1.1) at week 13. Notably, stent type affected time-dependent development of vessel injury, resulting in less vessel injury at week 13 with Misago than with the other stents (Fig. 2C). Moreover, vessel injury at weeks 4 and 13 was generally less in the proximal segment than in the other segments (Fig. 3C). These findings indicate that mid or distal segment is more susceptible to mechanical stress. According to coronary bare stenting studies [20–24], vessel enlargement and injury are correlated with NIH. Of note, the present study found that IEL enlargement is independently associated with NIH both at weeks 4 and 13, while vessel injury is independently associated with NIH only at week 13. These finding indicate that progressive IEL enlargement is a long-acting and progressive vessel injury is a late-acting contributor to NIH. Given that vessel wall can be affected by stent outward force, stent property of exerting greater self-expanding forces might be counterproductive in the femoropopliteal artery.

### Hallmark of vessel inflammation

The degree of vessel inflammation after coronary stenting is mild to moderate over time [12, 19, 25–27]. The present study found that vessel inflammation develops from moderate level (inflammation score: 1.8 with all 3 stents) at week 4 to severe level (inflammation score: 2.3 to 3.4) at week 13, indicating more serious vessel inflammation in the femoropopliteal artery than in the coronary artery. Notably, stent type affected time-dependent development of vessel inflammation, resulting in less vessel inflammation at week 13 with Misago than with the other stents (Fig. 2D). Also, because of the interaction effect of stent type on distribution of vessel inflammation, Misago had less inflammation in the proximal segment over time, while the other stents had comparable inflammation across all segments at week 13 (Fig. 3D). These findings indicate that development and distribution of vessel inflammation are diverse even among bare nitinol SESs. According to the previous coronary study with bare BESs (25), vessel inflammation is correlated with NIH. The present study found that vessel inflammation is independently associated with NIH both at weeks 4 and 13, indicating that vessel inflammation is a long-acting contributor to NIH. Therefore, suppression of vessel inflammation might assure the mitigation of NIH over time.

### Hallmark of eosinophils infiltration

Eosinophils can infiltrate as an allergic inflammation after coronary stenting with DESs rather than BESs [28]. However, no data are available regarding eosinophils infiltration after femoropopliteal stenting. In the present study, eosinophils infiltration at week 4 was comparable at a moderate level among the 3 stents. However because of the interaction effect of stent type, eosinophils infiltration decreased with Misago and increased with the other stents at week 13 as compared to week 4, resulting lower degree of eosinophils infiltration with Misago compared with the other stents (Fig. 2E). These findings indicate that time-dependent development of eosinophils infiltration is heterogeneous among bare nitinol SESs. According to the previous coronary stenting study, eosinophils infiltration was associated with NIH in patients with adverse events [28]. However, the present study found that eosinophils infiltration is not independently associated with NIH. Therefore, the role of allergic inflammation might be negligible in the neointimal process after implantation of bare nitinol SESs in the femoropopliteal artery.

### Hallmark of giant cells infiltration

Giant cell formation that is a fusion of several macrophages is recognized as the end-stage of foreign body reaction [29]. In the previous coronary stenting studies, the percentage of struts with giant cells was less than 20% over time [18, 27]. However, scanty data are available regarding giant cells infiltration after femoropopliteal stenting. In the present study, giant cells infiltration at week 4 was mild to moderate (the percentage of struts with giant cells: Misago 2.8%, Smart Flex 5.2%, and Innova 16.7%), which level appears to be consistent with that after coronary stenting. Intriguingly, because of the interaction effect of stent type, only Innova exhibited considerable development of giant cell infiltration at week 13, resulting in the highest degree of giant cells infiltration among the 3 stents (the percentage: Misago 4.3%, Smart Flex 12.9%, and Innova 40.8%) (Fig. 2F). These findings indicate that Innova can be characterized by outstanding chronic foreign body reaction as compared with the other stents. Moreover, the present study found that giant cells infiltration is independently associated with NIH at week 13, indicating that giant cells infiltration is a late-acting contributor to NIH. Hence, at the chronic stage, emphasis should be placed on improving femoropopliteal stent biocompatibility to mitigate NIH related to foreign body reaction in addition to a reaction to chronic outward force.

### Net lumen loss and future perspective of nitinol self-expanding stent platform

Stent scaffolding has been traditionally thought to be beneficial to secure the lumen by alleviating vessel constraint-induced stent recoil. In particular, SES can play a role in expanding the vessel increasingly over time. Thus, net lumen can be determined by the balance between increasing stent expansion and NIH. The present study confirmed that progressively increased neointima compensates for delayed expanding space inside the stent in the 3 stents. Also, as shown in Smart Flex, mean RVD was larger at weeks 4 and 13 than before stenting, indicating that peri-stent positive remodeling is potentially involved in the process of vascular remodeling. Importantly, the present study found that Misago preserves lumen area especially in the distal segment as compared with the other stents (Fig. 2H, 3H, 5, 6). Moreover, net lumen as assessed by MLD and %DS was eventually preserved only with Misago, but was lost with the other stents (Fig. 7). These findings indicate two types of vascular responses from the perspective of net lumen loss: one is that the advantage of less NIH outweighs the disadvantage of less vessel enlargement (that is rephrased as Misago type); the other is that the disadvantage of more NIH outweighs the advantage of more vessel enlargement (that is rephrased as Smart Flex or Innova type). Hence, given that Misago can be characterized by less fibrin deposition, vessel stress, and inflammatory reaction, further improvement in stent property and biocompatibility and novel drug-eluting technology are indispensable in terms of optimizing anti-proliferative drug and developing next-generation femoropopliteal DES platform.

### Limitations

First, although multiple disorders are involved in peripheral artery disease, this study was experimental in nature and stents were implanted in healthy swine femoropopliteal arteries. Thus, the findings might not be adapted to the clinical setting. Second, this study had a follow-up duration of 13 weeks for evaluating vascular responses. However, it remains unclear whether physical and pathological reactions to nitinol SESs can further change during the remote period. Third, the potential for contamination with industrial impurities on the stent surface might have affected vascular responses [30].

## Conclusions

Three bare nitinol SESs displayed a broad spectrum of vascular response. Increased fibrin deposition was an early-acting, progressive IEL enlargement and vessel inflammation were long-acting, and progressive vessel injury and giant cells infiltration were late-acting contributors to NIH. In the context of bare nitinol SES platform with less progressive mechanical stress and inflammatory reaction, the advantage of less NIH outweighed the disadvantage of less vessel enlargement, leading to net lumen preservation. These findings can contribute to optimize anti-proliferative drug and develop next-generation femoropopliteal DES platform.

## Supplementary Information

Below is the link to the electronic supplementary material.

Supplemental Fig. 1. Study flow chart file1 (TIF 1102 KB)

Supplemental Fig. 2. Representative angiograms (TIF 2428 KB)

Supplemental Fig. 3. Representative histological images. A. Fibrin deposition. Arrows indicate fibrin deposition surrounding the stent strut (elastin trichrome stain). B. Vessel injury. Arrows indicate ruptured internal and external elastic laminae (elastin trichrome stain). C. Vessel inflammation. Arrows indicate circumferentially extensive infiltration of inflammatory cells around the stent strut (hematoxylin and eosin stain). D. Eosinophils infiltration. Arrows indicate eosinophils infiltration around the stent strut (hematoxylin and eosin stain). E. Giant cells infiltration. Arrows indicate giant cells infiltration around the stent strut (hematoxylin and eosin stain) (TIF 8035 KB)
