# Supplementary figures and images for: Heterogeneous vascular response after implantation of bare nitinol self-expanding stents in the swine femoropopliteal artery
Source: Cardiovasc Interv Ther. 2022 Oct 18;38(2):210–22. doi: 10.1007/s12928-022-00889-5 (PMC10020252; doi:10.1007/s12928-022-00889-5)

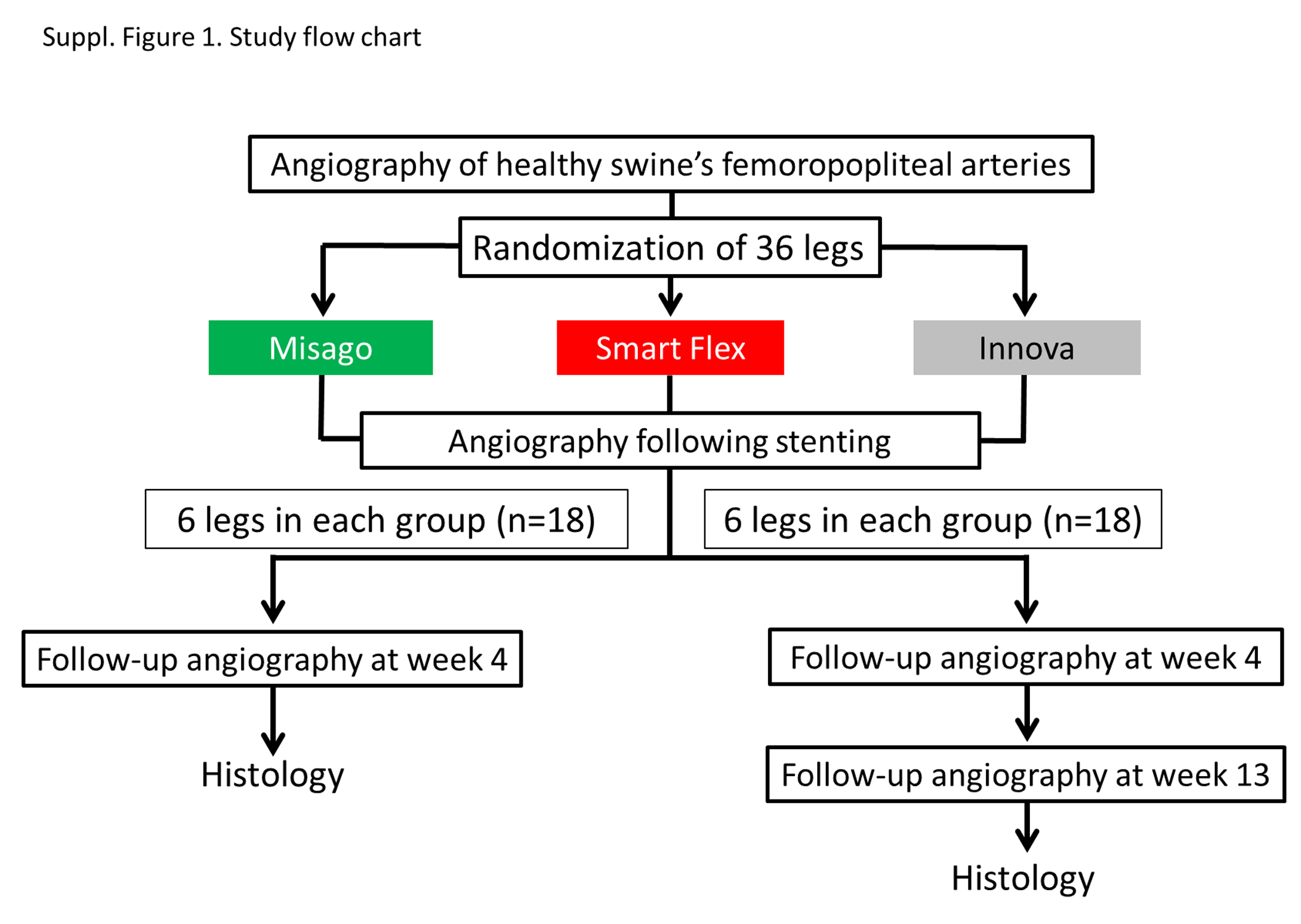

Supplement: Supplementary file 1 — Supplemental Fig. 1. Study flow chart file1 (TIF 1102 KB) [file 12928_2022_889_MOESM1_ESM.tif]

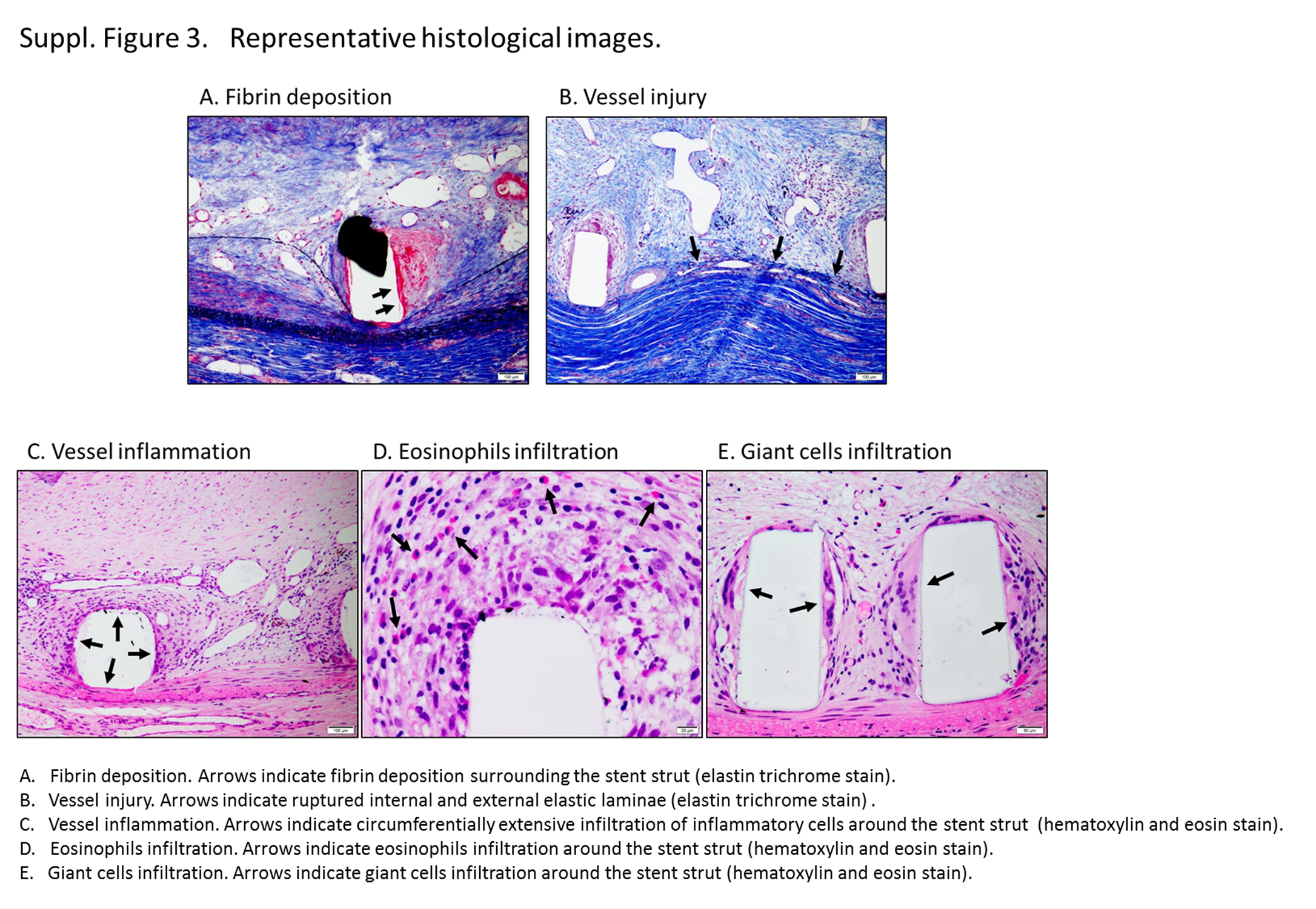

Supplement: Supplementary file 3 — Supplemental Fig. 3. Representative histological images. A. Fibrin deposition. Arrows indicate fibrin deposition surrounding the stent strut (elastin trichrome stain). B. Vessel injury. Arrows indicate ruptured internal and external elastic laminae (elastin trichrome stain). C. Vessel inflammation. Arrows indicate circumferentially extensive infiltration of inflammatory cells around the stent strut (hematoxylin and eosin stain). D. Eosinophils infiltration. Arrows indicate eosinophils infiltration around the stent strut (hematoxylin and eosin stain). E. Giant cells infiltration. Arrows indicate giant cells infiltration around the stent strut (hematoxylin and eosin stain) (TIF 8035 KB) [file 12928_2022_889_MOESM3_ESM.tif]
